# Development of oncolytic virotherapy: from genetic modification to combination therapy

**DOI:** 10.1007/s11684-020-0750-4

**Published:** 2020-03-07

**Authors:** Qiaoshuai Lan, Shuai Xia, Qian Wang, Wei Xu, Haiyan Huang, Shibo Jiang, Lu Lu

**Affiliations:** 1 Key Laboratory of Medical Molecular Virology (MOE/NHC/CAMS), School of Basic Medical Sciences and Shanghai Public Health Clinical Center, Fudan University, Shanghai, 200032 China; 2 Yantai Yuhuangding Hospital, Qingdao University, Yantai, 264000 China; 3 Lindsley F. Kimball Research Institute, New York Blood Center, New York, NY 10065 USA

**Keywords:** immunotherapy, oncolytic virus, genetic modification, immune checkpoint blockade, chimeric antigen receptor T cell

## Abstract

Oncolytic virotherapy (OVT) is a novel form of immunotherapy using natural or genetically modified viruses to selectively replicate in and kill malignant cells. Many genetically modified oncolytic viruses (OVs) with enhanced tumor targeting, antitumor efficacy, and safety have been generated, and some of which have been assessed in clinical trials. Combining OVT with other immunotherapies can remarkably enhance the antitumor efficacy. In this work, we review the use of wild-type viruses in OVT and the strategies for OV genetic modification. We also review and discuss the combinations of OVT with other immunotherapies.

## Introduction

Immunotherapy is an important antitumor therapy that involves stimulating or enhancing host antitumor immune response to kill and clear tumor cells [1,2]. Oncolytic virotherapy (OVT) is a new antitumor immunotherapy that uses natural or genetically modified viruses to selectively infect and kill tumor cells [3]. OVT has various advantages over current antitumor therapies. Oncolytic viruses (OVs) can selectively replicate in tumor cells with high safety [4] and carry foreign genes, such as therapeutic and immunostimulatory genes [5] for specific expression at tumor sites [6]. Some OVs can cross the blood–brain barrier (BBB) to kill brain tumor cells, such as reovirus [7] and parvovirus H-1 (H-1PV) [8], and OVs can turn “cold” tumors into “hot” tumors, thereby increasing the cellular sensitivity to other immunotherapies [9,10]. To date, many OVs, including DNA viruses, such as adenovirus (AdV) [11,12], vaccinia virus (VACV) [13,14], herpesvirus [15,16], and parvovirus [17] and RNA viruses, such as reovirus [18,19], Newcastle disease virus (NDV) [20], and measles virus (MV) [21] (Table 1) have been evaluated for cancer treatment (Fig. 1).

In the last century, scientists found that some viruses, such as NDV [32], MV [33], and parvovirus [34], can naturally kill tumor cells. However, during this period, OVs were derived from wild-type or naturally attenuated virus strains, leaving considerable room for improvement in their safety and antitumor effects [35,36]. In the 1990s, the emergence of recombinant DNA technology accelerated the development of OVT. Genetically modified OVs showed enhanced tumor targeting and killing efficiency against tumor cells with negligible damage to normal cells. The first genetically modified OV was herpes simplex virus (HSV)-1 developed by Martuza *et al.* in 1991 [37,38]. Oncorine (H101), a genetically modified oncolytic adenovirus (OAd), was approved by the State Food and Drug Administration (SFDA, now the National Medical Products Administration (NMPA)) in 2005 for the treatment of nasopharyngeal carcinoma in China [39,40]. Talimogene laherparepvec (T-VEC; brand name Imlygic) was approved by the US Food and Drug Administration (FDA) and European Medicines Agency (EMA) for the treatment of advanced melanoma in 2015 [41,42].

Researchers have recently attempted to combine OVT with other antitumor therapies, including chemotherapy

*These authors contributed equally to this work.

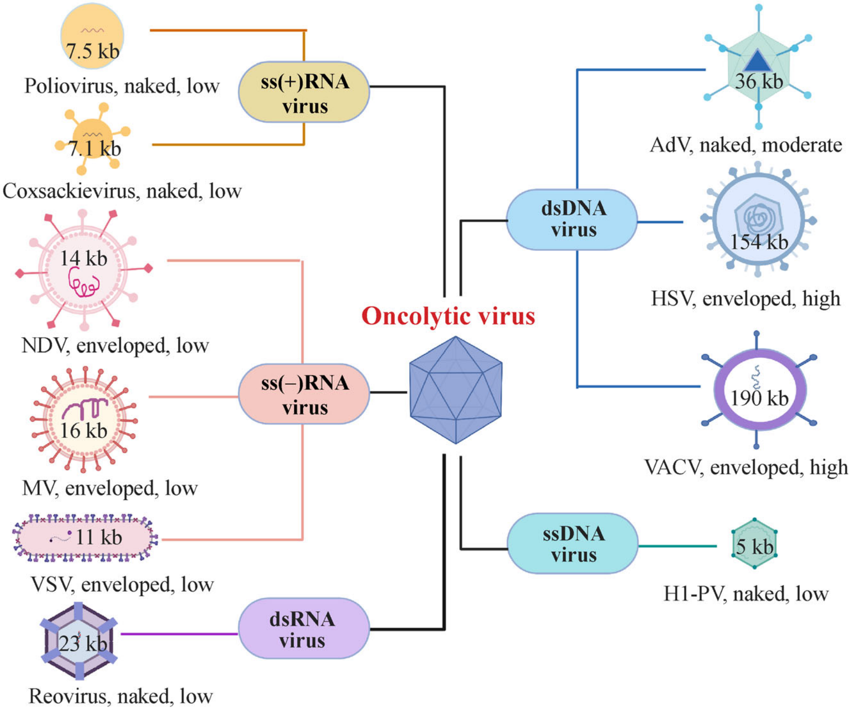

**Fig. 1** Schematic of some oncolytic viruses for cancer treatment. Many viruses, including double-stranded DNA viruses, such as AdV, herpes simplex virus (HSV), and VACV; single-stranded DNA viruses, such as parvovirus; negative-sense single-stranded RNA viruses, such as Newcastle disease virus (NDV), measles virus (MV), and vesicular stomatitis virus (VSV); positive-sense single-stranded RNA viruses, such as poliovirus and coxsackievirus; and double-stranded RNA viruses, such as reovirus, have been used in cancer treatment. The terms naked/enveloped are used to describe types of virus; high/moderate/low describes the genomic capacities of OVs.

[43–45], radiotherapy [46,47], small molecules [48,49], and other immunotherapies [50,51], to achieve increased therapeutic effects against tumors. Among these treatments, the combination of OVT with other immunotherapies, especially regimens involving immune checkpoint inhibitors (ICIs) and chimeric antigen receptor-engineered T cell (CAR-T cell) immunotherapies, has shown promise as a cancer treatment [51,52]. OVs can "heat up" tumors, thus enhancing their sensitivity to ICIs [53]. OVs can also piggyback CAR-T cells in a manner that enables them to overcome the immunosuppressive tumor microenvironment (TME) and increase their antitumor activity against solid tumors [54]. Recently, natural OVs have been continuously identified and widely applied in OVT. The renewed development of genetically modified OVs with enhanced tumor-targeting ability, antitumor efficacy, and safety has further promoted the development of OVT. In this work, we review the applications of wild-type viruses and the modifications for improving the tumor-targeting ability, antitumor efficacy, and safety of OVs. We also review and discuss the important research directions for OVT combination with other immunotherapies, including immune checkpoint blockade (ICB) and CAR-T cell therapies.

## Wild-type OVs in oncolytic virotherapy

Some wild-type OVs have a natural tropism for tumor cells based on their capacity to recognize highly expressed receptors on tumor cell surfaces or based on abnormal pathways or products in tumor cells, such as a defect in the interferon (IFN) signaling pathway and the activated Ras pathway. Among them, several representative OVs, such as coxsackievirus [55], parvovirus [56], and reovirus [57], have been widely reported.

Coxsackievirus A21 (CVA21) is a promising OV that depends on highly expressed receptor molecules on the surface of tumor cells, intercellular adhesion molecule-1 (ICAM-1, also named CD54), and decay-accelerating factor to preferentially enter, replicate in, and kill tumor cells. CVA21 exhibited antitumor efficacy in multiple cancers, including melanoma, prostate cancer, and multiple myeloma [30]. A CVA21-based OV (CAVATAK®) has been assessed in clinical trials for the treatment of malignant melanoma, bladder cancer [58], and uveal melanoma with liver metastases [59]. The results revealed that CAVATAK® has a good safety profile and can stimulate systemic antitumor immunity for the treatment of patients with melanoma [60]. CAVATAK® can also

**Table 1** Characteristics of DNA virus- and RNA virus-derived OVs

| Virus type | Virus | Family | Natural host | Receptor | Replication site | Common used virus strain or type | References |
|---|---|---|---|---|---|---|---|
| DNA virus | Adenovirus | Adenoviridae | Human | CAR, integrin | Nucleus, cytoplasm | Ad5 | [22] |
| | Vaccinia virus | Poxviridae | Human | Unknown | Cytoplasm | Lister, Wyeth, WR | [23] |
| | Herpesvirus | Herpesviridae | Human | HVEM, nectin | Nucleus, cytoplasm | HSV-1 | [24] |
| | Parvovirus | Parvoviridae | Rat | SARs | Nucleus, cytoplasm | H1-PV | [25] |
| RNA virus | Measles virus | Paramyxoviridae | Human | CD46, SLAM | Cytoplasm | Edmonston vaccine strain | [26] |
| | Newcastle disease virus | Paramyxoviridae | Chicken | Unknown | Cytoplasm | HUJ, MTH-68/H | [27] |
| | Vesicular stomatitis virus | Rhabdoviridae | Pigs, cattle, horses | LDLR | Cytoplasm | Recombinant Indiana strain | [28] |
| | Poliovirus | Picornaviridae | Human | CD155 | Cytoplasm | Sabin vaccine strain | [29] |
| | Coxsackievirus | Picornaviridae | Human | ICAM-1, DAF | Cytoplasm | CVA21, CVB3 | [30] |
| | Reovirus | Reoviridae | Human | Unknown | Cytoplasm | Reovirus-serotype 3 | [31] |

CAR, coxsackie adenovirus receptor; HVEM, herpesvirus entry mediator; SARs, sialic acid residues; SLAM, signaling lymphocytic activation molecule; LDLR, low-density lipoprotein receptor; ICAM-1, intercellular adhesion molecule 1; DAF, decay accelerating factor.

induce immune cells to infiltrate the TME, indicating that its use in combination with other antitumor immunotherapies can increase antitumor efficacy.

Wild-type H-1PV exhibits tumor selectivity, mainly depending on tumor-specific abnormal replication and transcription factors and the defective type I IFN-mediated antiviral pathway in tumor cells [17]. H-1PV nonstructural protein 1 (NS1) is important for promoting tumor cell death. Preclinical studies suggested that H-1PV can effectively kill various tumor cells [56], including pancreatic ductal adenocarcinoma cells and glioma cells [61]. In particular, ParvOryx (wild-type H-1PV) showed an excellent safety profile in phase I/IIa clinical trials [8]. Wild-type reovirus can also selectively replicate in tumor cells in association with the abnormally activated Ras pathway in tumor cells [62]. Reovirus can cross the BBB to kill brain tumor cells through intravenous administration [7]. Reolysin® (pelareorep) is a formulation of the reovirus serotype 3 Dearing strain and is being assessed in clinical trials for the treatment of multiple myeloma [63], pancreatic adenocarcinoma [64], and melanoma [65]. Reolysin® has received an orphan drug designation from the US FDA and EMA for the treatment of gastric and pancreatic cancers [31]. In the treatment of multiple myeloma, the single use of Reolysin® did not show obvious antitumor activity [66]. However, when Reolysin® was combined with chemotherapy, such as carboplatin and paclitaxel, it exhibited improved therapeutic efficacy with an objective response rate (ORR) of up to 21% in the treatment of malignant melanoma [19].

Many alphaviruses, such as Semliki Forest virus (SFV) [67,68], Sindbis virus (SINV) [69], and M1, exhibit antitumor activity. M1 is a recently identified Getah-like alphavirus from culicine mosquitoes [70,71]. M1 has tumor selectivity related to zinc-finger antiviral protein (ZAP) deficiency in tumor cells and therefore can selectively replicate in tumor cells and induce apoptosis of the infected tumor cells by inducing irreversible endoplasmic reticulum (ER) stress [72]. M1 showed high tumor cell selectivity and tumor-killing activity during *in vitro* and *in vivo* experiments [73]. Moreover, its antitumor activity can be considerably increased by targeted inhibitors, such as valosin-containing protein (VCP) inhibitor and DNA-dependent kinase (DNA-PK) inhibitor [74,75], suggesting that combining M1 with these inhibitors is an important strategy.

## Genetic modifications of OVs

Genetic modification has accelerated the development of OVT [76]. In 1991, the first study reported on a genetically engineered OV, that is, an HSV with thymidine kinase (TK) deletion [38]. Many genetically modified OVs have been evaluated in clinical trials, as shown in Table 2; such OVs include AdV, VACV, and HSV. OVs mainly genetically modified through the deletion of virulence genes to improve the safety and insertion of foreign genes and improve antitumor efficacy or tumor targeting ability of OVs [77]. The genetic modifications of OVs can be classified into four categories according to purpose: (1) enhancing tumor targeting, (2) improving the safety of OVs, (3) increasing antitumor efficacy, and (4) monitoring replication status of OVs *in vivo* [78] (Fig. 2).

## Enhancing tumor targeting

The tumor tropism of OVs is an important feature to ensure that they selectively replicate in and specifically kill tumor cells [79]. Wild-type OVs generally depend on tumor-specific cellular receptors or abnormal intracellular pathways and products to enter and selectively replicate in tumor cells and therefore can be optimized through genetic modifications to enhance their tumor targeting. Many genetically engineered OVs with enhanced tumor cell targeting ability have been developed on the basis of the tumor-specific high expression of certain receptors and the abnormal intracellular signaling pathways and metabolic status [80].

### *Engineered tumor tropism targeting specific tumor surface receptors*

The nonspecificity, low affinity, or low expression of natural viral receptors on tumor cells can limit the application of wild-type OVs. Thus, OVs should be genetically modified to increase their binding affinity to specifically expressed receptors on tumor cells and increase their efficiency in entering and targeting tumor cells.

Inserting targeting peptides can improve the entry efficiency of OVs [81]. For example, adenovirus type 5 (Ad5) is a widely used OV vector, but its receptor, coxsackie adenovirus receptor (CAR), is not highly expressed in many tumor cells [22]; this phenomenon leads to the low entry efficiency of OAd. Through genetic engineering, an arginine–glycine–aspartic acid (RGD) motif was inserted into the fiber knob domain of Ad5 to generate a newly modified virus, which no longer depends on CAR to enter tumor cells and instead relies on integrins that are highly expressed on tumor cells, to enter tumor cells [82]. Other studies inserted the fiber knob domain derived from adenovirus type 3 (Ad3) into the backbone of Ad5 (also named serotype switching) to allow its entrance to the tumor cells by utilizing the highly expressed Ad3 receptor (desmoglein 2 as the primary receptor) on tumor cells to enter the tumor cells [79]. A similar modification involves inserting the fiber knob domain of adenovirus type 35 (Ad35) into the backbone of Ad5, allowing the OVs to utilize the Ad35 receptor (CD46 as the primary receptor) to enter tumor cells [81].

Some envelope glycoproteins (G) from other virus families can be inserted to improve tumor cell targeting. Betancourt *et al.* [83] reported replacing the G protein of vesicular stomatitis virus (VSV) with human immunodeficiency virus type 1 (HIV-1) gp160 to generate a new OV (VSV-gp160G); this method abandons the natural tissue tropism of VSV and specifically targets the new receptor, CD4. VSV-gp160G does not damage normal CD4 T cells; however, it exhibits potent killing activity against CD4-expressing tumor cells, such as adult T cell leukemia/lymphoma cell lines, in association with defective antiviral immune pathways in these tumor cells. A gene encoding a single-chain antibody (scAb) targeting human epidermal growth factor receptor 2 (HER2) was incorporated into HSV-1, making it fully targeted to tumor cells expressing HER2, and HER2-negative cells were left unharmed [84–86]. This modification also enhanced safety. Insertions of scAb targeting tumor-surface antigens, such as epithelial cell adhesion molecule (EpCAM) [87] and human carcinoembryonic antigen (CEA) [88], have been reported to improve the tumor targeting of OVs. Shibata *et al.* [87] reported the insertion of the scAb targeting EpCAM (scEpCAM) into the HSV-1 genome. The engineered virus is highly specific to the EpCAM expressed on the surface of tumor cells to enter tumor cells; it can efficiently and specifically kill tumor cells expressing EpCAM. This strategy can improve the tumor targeting of OVs. However, these OVs have not yet entered clinical trials, and their clinical efficacy needs to be further confirmed.

### *Engineered tumor tropism targeting the intracellular abnormalities of tumor cells*

The various signaling pathways and the metabolic status of tumor cells are altered compared with those of normal cells [89]. Many features of tumor cells can be utilized by OVs to generate tumor selectivity [90]; such features include defective antiviral pathways, such as a defective type I IFN signaling pathway [91]; the loss of expression of tumor suppressor genes, such as loss of the retinoblastoma gene [92]; and elevated signaling pathways, such as a transformed Ras pathway [93]. Therefore, targeting these abnormal pathways or products in tumor cells, deleted virulence factors and inserted tumor-specific promoters or microRNA-targeting sequences (miRNA-TSs) of OVs can regulate OVs specifically replicating in tumor cells [94,95]. Deletion of virulence factors can improve the safety of OVs, which is discussed in the next section on the safety of genetically modified OVs. This section introduces the strategies for inserting tumor-specific promoters or miRNA-TSs to improve the tumor targeting of OVs.

Insertion of tumor-specific promoters, such as the human telomerase reverse transcriptase (hTERT) promoter [96] and the prostate-specific antigen (PSA) promoter [97], can improve the tumor selectivity of OVs [3], which will, in turn, drive the expression of viral genes in tumor cells

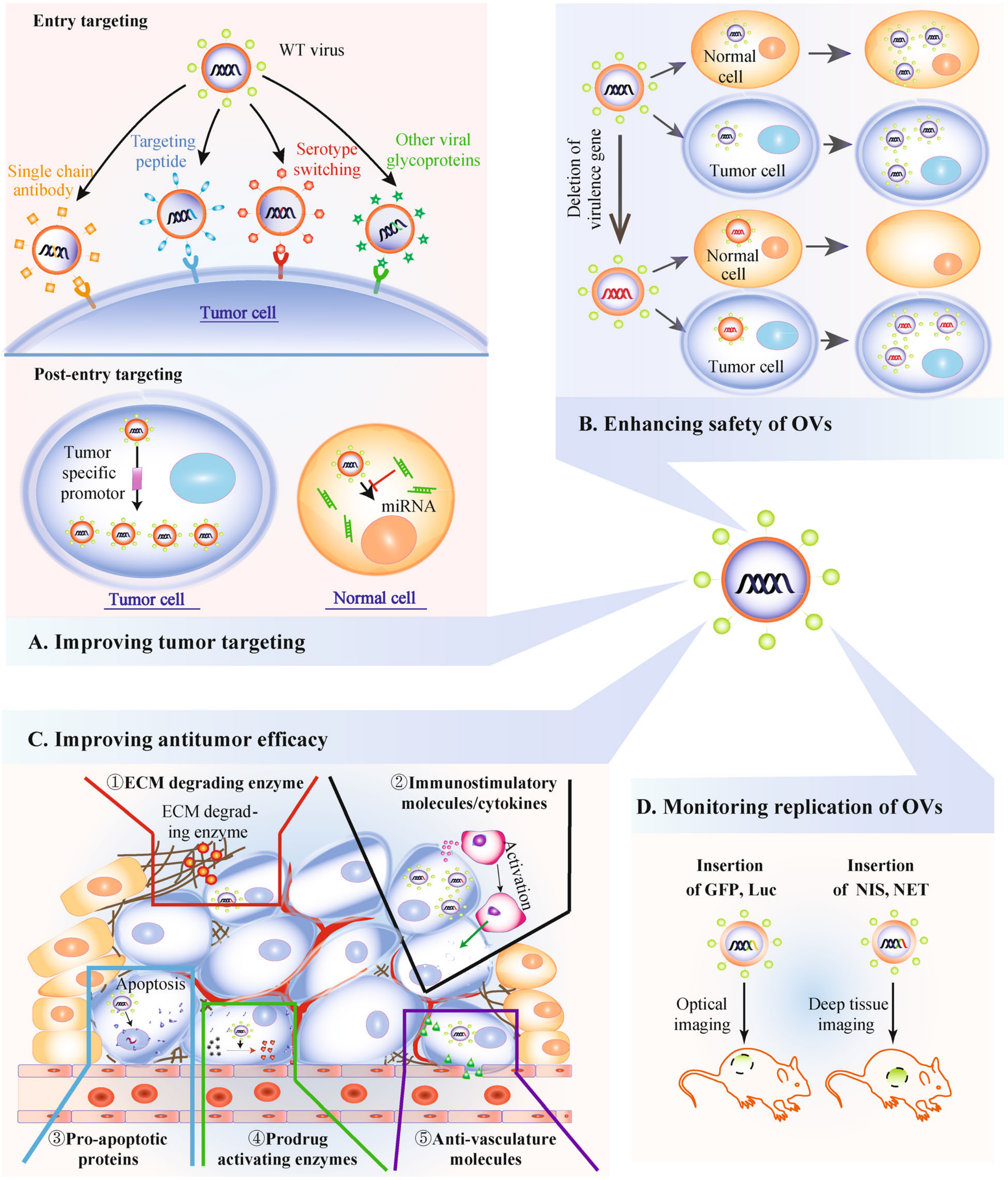

**Fig. 2** Genetic modifications of OVs. (A) Genetic modifications for improving tumor targeting of OVs. Entry targeting: serotype switching and insertion of a tumor-targeting peptide, glycoproteins from other viruses, and single-chain antibodies (scAb) targeting tumor-associated antigens can allow OVs to target tumor surface molecules and enter tumor cells. Post-entry targeting: insertion of tumor-specific promotors can promote virus replication in tumor cells, while insertion of miRNA target sequences can restrict virus replication in normal cells. (B) Genetic modifications that enhance OV safety. Deletion of virulence genes can reduce the risk of OV infections of normal cells and enhance safety. (C) Genetic modifications to augment the antitumor efficacy of OVs. Antitumor efficacy can be augmented by inserting foreign genes, such as genes encoding immunostimulatory molecules/cytokines, suicide genes (pro-apoptotic proteins and prodrug-activating enzymes), extracellular matrix (ECM)-degrading enzymes, and anti-vasculature molecules. (D) Genetic modifications to monitor OV replication. Reporter genes, such as green fluorescent protein (GFP), *Renilla* luciferase (Rluc), sodium–iodide symporter (NIS), and human norepinephrine transporter (NET), can be inserted to monitor OV replication.

**Table 2** Oncolytic viruses in clinical trials

| Virus | Name of OVs | Genetic modification | Conditions | Phase | NCT Number | Status | References |
|---|---|---|---|---|---|---|---|
| HSV | T-VEC | ICP34.5 and ICP47 deletion; GM-CSF insertion | Advanced melanoma | Phase 1 | NCT03747744 | Recruiting | [170–172] |
| | | | Breast cancer | Phase 1/2 | NCT02779855 | Recruiting | |
| | | | Melanoma | Phase 2 | NCT00289016 | Completed | |
| | | | Melanoma | Phase 3 | NCT01368276 | Completed | |
| | | | Melanoma | Phase 3 | NCT00769704 | Completed; DRR: 36.1% (T-VEC-treated patients) versus 3.8% (GM-CSF treated patients) | |
| | HSV-1716 | ICP34.5 deletion | Glioma | Phase 1 | NCT02031965 | Terminated | |
| | | | Rhabdomyosarcoma Osteosarcoma | Phase 1 | NCT00931931 | Completed | |
| | G207 | ICP34.5 deletion; UL39 disruption | Glioma | Phase 1/2 | NCT00028158 | Completed | |
| | | | Brain tumors | Phase 1 | NCT03911388 | Not yet recruiting | |
| | | | Malignant glioma | Phase 1 | NCT02457845 | Recruiting | |
| | M032 | ICP34.5 deletion; IL12 insertion | Glioblastoma multiforme | Phase 1 | NCT02062827 | Recruiting | |
| | HF10 | HSV-HF strain | Melanoma | Phase 2 | NCT03259425 | Active, not recruiting | [173] |
| | | | Solid tumors with superficial lesions | Phase 1 | NCT02428036 | Completed | |
| | | | Pancreatic cancer | Phase 1 | NCT03252808 | Active, not recruiting | |
| | | | Melanoma | Phase 2 | NCT03153085 | Completed | |
| AdV | LOAd703 | CD40L and 4-1BBL insertion; Δ24 in E1A | Pancreatic adenocarcinoma | Phase 1/2 | NCT03225989 | Recruiting | [11,156,174] |
| | | | Pancreatic cancer | Phase 1/2 | NCT02705196 | Recruiting | |
| | VCN-01 | Hyaluronidase and RGD insertion; Δ24 in E1A | Metastatic solid tumor | Phase 1 | NCT02045602 | Active, not recruiting | |
| | | | Pancreatic adenocarcinoma | Phase 1 | NCT02045589 | Completed | |
| | | | Retinoblastoma | Phase 1 | NCT03284268 | Recruiting | |
| | DNX-2401 (Delta-24-RGD) | RGD insertion; Δ24 in E1A | Glioblastoma | Phase 1 | NCT03896568 | Recruiting | |
| | | | Brain stem glioma | Phase 1 | NCT03178032 | Recruiting | |
| | | | Brain cancer | Phase 1 | NCT00805376 | Completed; 20% of patients survived>3 years | |
| | CG0070 | GM-CSF insertion; E3 deletion | Bladder cancer | Phase 2 | NCT02143804 | Withdrawn | |
| | | | Bladder cancer | Phase 2 | NCT02365818 | Completed; overall 47% CR rate at 6 months for all patients and 50% for patients with CIS | |
| | ONCOS-102 (CGTG-102) | Type 3 knob and GM-CSF insertion; Δ24 in E1A | Malignant solid tumor | Phase 1 | NCT01598129 | Completed | |
| | | | Advanced or unresectable melanoma progressing after PD1 blockade | Phase 1 | NCT03003676 | Recruiting | |
| | OBP-301 | hTERT promoter insertion | Hepatocellular carcinoma | Phase 1 | NCT02293850 | Recruiting | [175] |
| | | | Melanoma | Phase 2 | NCT03190824 | Active, not recruiting | |
| | | | Esophagogastric adenocarcinoma | Phase 2 | NCT03921021 | Recruiting | |
| | | | Esophageal cancer | Phase 1 | NCT03213054 | Recruiting | |

(*Continued*)

| Virus | Name of OVs | Genetic modification | Conditions | Phase | NCT Number | Status | References |
|---|---|---|---|---|---|---|---|
| VACV | JX-594 (Pexa-Vec) | TK deletion; GM-CSF insertion | Melanoma | Phase 1/2 | NCT00429312 | Completed | [111,148,176, 177] |
| | | | Ovarian cancer | Phase 2 | NCT02017678 | Withdrawn | |
| | | | Melanoma | Phase 1 | NCT00625456 | Completed | |
| | | | Renal cell carcinoma | Phase 1 | NCT03294083 | Recruiting | |
| | | | Colorectal cancer | Phase 1/2 | NCT03206073 | Recruiting | |
| | | | Hepatocellular carcinoma | Phase 2 | NCT00554372 | Completed; intrahepatic mRECIST<br>Disease control rate at week 8 was 46% overall | |
| | GL-ONC1 | Ruc-GFP, β-glucuronidase, and β-galactosidase insertion<br>TK and haemagglutinin disruption | Solid organ cancers | Phase 1 | NCT02714374 | Active, not recruiting | |
| | | | Peritoneal carcinomatosis | Phase 1/2 | NCT01443260 | Completed; efficient intraperitoneal infection in eight patients (8/9) | |
| | | | Cancer of head and neck | Phase 1 | NCT01584284 | Completed; combinations of GL-ONC1 with radiotherapy and chemotherapy: 1-year (2-year) PFS and OS were 74.4% (64.1%) and 84.6% (69.2%), respectively | |
| | | | Advanced cancers (solid tumors) | Phase 1 | NCT00794131 | Completed | |
| | TG6002 | FCU1 insertion; J2R and I4L deletion | Glioblastoma | Phase 1/2 | NCT03294486 | Recruiting | |
| | | | Colon cancer | Phase 1/2 | NCT03724071 | Recruiting | |
| MV | MV-NIS | NIS insertion | Multiple myeloma | Phase 2 | NCT02192775 | Recruiting | [169] |
| | | | Malignant ovarian tumor | Phase 2 | NCT02364713 | Recruiting | |
| | MV-CEA | CEA insertion | Glioblastoma | Phase 1 | NCT00390299 | Active, not recruiting | |
| | | | Ovarian epithelial cancer or primary peritoneal cancer | Phase 1 | NCT00408590 | Completed | |
| VSV | VSV-IFNβ-NIS | IFNβ and NIS insertion | Malignant solid tumor | Phase 1 | NCT02923466 | Recruiting | [178] |
| | | | Hepatocellular carcinoma | Phase 1 | NCT03647163 | Recruiting | |
| Poliovirus | PVSRIPO | Replace IRES of poliovirus with IRES from HRV2 | Malignant glioma | Phase 1 | NCT03043391 | Recruiting | [179,180] |
| | | | Glioblastoma | Phase 1 | NCT01491893 | Active, not recruiting; OS among the patients who received PVSRIPO reached a plateau of 21% (95% confidence interval, 11 to 33) at 24 months | |
| | | | Malignant glioma | Phase 2 | NCT02986178 | Recruiting | |
| | | | Melanoma | Phase 1 | NCT03712358 | Recruiting | |
| Maraba virus | MG1-MAGEA3 | MAGEA3 insertion | Metastatic melanoma | Phase 1 | NCT03773744 | Not yet recruiting | [181] |
| | | | Non-small cell lung cancer | Phase 1/2 | NCT02879760 | Active, not recruiting | |
| | MG1-E6E7 | E6, E7 antigens insertion | HPV-associated cancers | Phase 1 | NCT03618953 | Active, not recruiting | |

(*Continued*)

| Virus | Name of OVs | Genetic modification | Conditions | Phase | NCT Number | Status | References |
|---|---|---|---|---|---|---|---|
| Coxsackievirus | CAVATAK® | None (wild type) | Uveal melanoma with liver metastases | Phase 1 | NCT03408587 | Completed | [30] |
| | | | Malignant melanoma | Phase 2 | NCT01227551 | Completed | |
| Reovirus | Pelareorep (Reolysin®) | None (Wild type) | Malignant glioma | Phase 1 | NCT00528684 | Completed | [182] |
| | | | Osteosarcoma | Phase 2 | NCT00503295 | Completed | |
| | | | Bladder carcinoma | Phase 1 | NCT02723838 | Withdrawn | |
| | | | Pancreatic adenocarcinoma | Phase 1 | NCT02620423 | Completed | |
| | | | Pancreatic adenocarcinoma | Phase 2 | NCT01280058 | Completed; PFS: 4.9 months (paclitaxel/ carboplatin + pelareorep) versus 5.2 months (paclitaxel/carboplatin) | |
| | | | Head and neck cancers | Phase 3 | NCT01166542 | Completed | |
| Parvovirus H-1 | ParvOryx | None (Wild type) | Glioblastoma multiforme | Phase 1/2 | NCT01301430 | Completed; H-1PV treatment was safe and can extend median survival | [183,184] |
| | | | Pancreatic cancer | Phase 1/2 | NCT02653313 | Completed | |
| NDV | NDV | None (Wild type) | Glioblastoma | Phase 1/2 | NCT01174537 | Withdrawn | [20,185] |
| | | | Metastatic cancer | Phase 2 | NCT00348842 | Withdrawn | |

Clinical information from https://www.clinicaltrials.gov/.
OS, overall survival; PFS, progression-free survival; ORR, objective response rate; mRECIST, modified response evaluation criteria in solid tumors; MAGEA3, melanoma-associated antigen 3; IRES, internal ribosomal entry site; HRV2, human rhinovirus type 2; NIS, sodium/iodide symporter; CEA, carcinoembryonic antigen.

but not in normal cells. As an example, hTERT is highly expressed in most tumor cells but absent in normal cells [98]; hence, the inserted hTERT promoter in OVs can initiate viral gene expression and replication in tumor cells with high hTERT expression [99]. OBP-301, an OAd with an inserted hTERT promoter, can replicate in tumor cells specifically, such as renal cell carcinoma cells and prostate cancer cells [100].

Different from the mechanisms of action of inserted tumor-specific promoters, inserted miRNA-TSs can lead to the viral RNA degradation through miRNAs in normal tissues; therefore, engineered OVs can specifically replicate in tumor cells with low miRNA expression [101,102]. For example, targeting downregulated miRNA-7 in glioblastoma multiforme (GBM), researchers constructed a miRNA-7-sensitive oncolytic MV that specifically infects glioma cells without damaging normal cells [103].

## Improving the safety of OVs

The safety of OVs is important for their clinical use. Some natural viruses have the potential to kill tumor cells but can also damage normal cells. Numerous studies have suggested that the deletion of some genes necessary for viral replication in normal cells or virulence genes can improve OV safety in normal cells and maintain their oncolytic capacities; such genes include TK in oncolytic VACV [104,105] and ICP34.5 in oncolytic HSV-1 [106,107].

The viral products encoded by such genes are required for the replication of some OVs in normal cells, and they are abundant in tumor cells. Therefore, OVs can utilize tumor cell products to selectively replicate, but they cannot replicate in normal cells because relevant products are lacking. For example, TK, an important enzyme for nucleotide synthesis, is highly expressed in tumor cells [108] but only expressed at a low level in normal cells. TK encoded by VACV is essential for viral replication in normal cells. The deletion of the TK gene is an important genetic modification of oncolytic VACV [109,110] to improve its safety. Oncolytic VACV with TK deletion can replicate in tumor cells but not in normal cells. Therefore, TK deletion can increase the safety of oncolytic VACV. Some oncolytic VACVs, such as GL-ONC1 and Pexa-Vec (JX-594), currently being assessed in clinical trials have TK deletions. GL-ONC1 has been assessed in clinical trials for the treatment of patients with peritoneal carcinomatosis (phase I/II) or cancer of the head and neck (phase I). Phase I clinical trial of GL-ONC1 for the treatment of peritoneal carcinomatosis [13,111] showed its improved safety but also the emergence of minor adverse events. Phase II clinical trial results for Pexa-Vec showed that it could improve the survival of patients with liver cancer [14]; thus, this OV has now entered phase III clinical trials for the treatment of hepatocellular carcinoma.

Wild-type HSV-1 can cause latent infections in normal neurons, but the deletion of its neurovirulence factor (ICP34.5) can enhance its safety [112]. ICP34.5 antagonizes protein kinase R (PKR)-mediated immune response and is essential for infecting neurons. Oncolytic HSV with ICP34.5 deletion cannot replicate in neurons [24]. Given that PKR is usually downregulated in tumor cells, the virus can replicate normally in these tumor cells. HSV-1716, G207, and T-VEC are oncolytic HSVs with ICP34.5 deletions. T-VEC has been approved by the US FDA for the treatment of melanoma [113]. In a recent study, the ORR among patients with in-transit melanoma metastasis treated with T-VEC was 40.7% [114].

Zika virus (ZIKV) was recently found to have oncolytic properties [115], but ZIKV infections in children can cause serious consequences [116,117]. Wild-type ZIKV could infect glioblastoma stem cells (GSCs; an important target for glioblastoma treatment) and slow down the growth of tumors, but wild-type ZIKV also attacks normal nerve cells [115]; hence, its safety should be further improved. A live-attenuated ZIKV vaccine (ZIKV-LAV) candidate [118] with 10 nucleotide deletions in the 3′ untranslated region in the ZIKV genome can maintain its oncolytic activity with increased safety and gene stability [119,120]. In preclinical research, ZIKV-LAV effectively inhibited GSC growth and improved the survival of experimental animals compared with the mock control group. The median survival time of mice transplanted with 387 GSC cells was extended from 30 days to 48 days, while the median survival time of mice transplanted with 4121 GSC cells increased from 31 days to 53 days [119].

## Augmenting antitumor efficacy

OVs can kill tumor cells through the following mechanisms: selectively replicating in tumor cells and causing the oncolysis of tumor cells [121]; inducing the immunogenic cell death of tumor cells, thereby releasing damage-associated molecular pattern molecules, tumor-associated antigens, and pathogen-associated molecule patterns that stimulate antitumor immunity to kill other tumor cells [91]; and destroying tumor vasculature to indirectly kill tumor cells [122,123]. Insertion of genes coding for immunostimulatory molecules/cytokines, suicide genes, ECM-degrading enzymes, and anti-vasculature molecules can improve the antitumor efficacy of OVs [76,124,125].

### *Immunostimulatory molecules/cytokines*

OVs induce systemic antitumor immunity, and many cytokines, such as granulocyte-macrophage colony-stimulating factor (GM-CSF) and interleukin (IL)-12 can increase the antitumor immunity. Thus, many OVs have been armed with immunostimulatory cytokines to improve their therapeutic efficacy [126]. For example, GM-CSF can recruit antigen-presenting cells (APCs) and promote the presentation of tumor antigens [78]. Various OVs armed with GM-CSF, including T-VEC (HSV-1), Pexa-Vec (VACV), and CG0070 (AdV), have been evaluated in clinical trials. IL-12 can stimulate IFN-$\gamma$ production, enhance the effect of T cells, and promote antitumor activity [127]. M032 is an oncolytic HSV expressing IL-12 [128] and is being assessed in the clinical trial for the treatment of GBM (the results of which have not yet been reported). Other cytokines, such as IL-2 [129] and IL-15 [130], can also be inserted to improve antitumor efficiency. However, some secreted cytokines have systemic toxicity [131]. Liu *et al*. [132] have developed a new strategy to solve the side effects of the systemic administration of cytokines. They used membrane-bound IL-2, instead of secretory IL-2, to construct an oncolytic VACV that expresses membrane-bound IL-2 (named vvDD-IL-2-RG). Compared with the oncolytic VACV- expressing secretory IL-2, vvDD-IL-2-RG did not compromise antitumor activity and its toxicity was remarkably reduced.

Inserting other immunostimulatory molecules, such as the costimulatory molecules CD40 ligand (CD40L) and 4-1BB ligand (4-1BBL), has also enhanced the antitumor effects of OVs [133,134]. CD40 and 4-1BB belong to the tumor necrosis factor receptor (TNFR) superfamily. CD40 and 4-1BBL expressed on the surface of APCs and other cells can recognize and bind CD40L and 4-1BB on the surface of T cells, respectively, enhancing the ability of APCs to present antigens and promote T cell activation [135]. The recombinant adenovirus LOAd703 simultaneously expresses the immunostimulatory molecules CD40L and 4-1BBL [136], resulting in the effective activation of T cells through the CD40 and 4-1BB signaling pathways, respectively; as such, they can enhance antitumor immunity. LOAd703 has entered clinical trials for the treatment of pancreatic cancer [137] but the results have not yet been reported.

### *Suicide gene*

Suicide gene therapy includes two strategies: expressing pro-apoptotic proteins or toxin proteins to kill tumor cells directly and using prodrug-activating systems to kill tumor cells indirectly [138]. Engineered OVs can express pro-apoptotic proteins or toxin proteins to directly kill tumor cells, such as tumor necrosis factor-related apoptosis-inducing ligand (TRAIL) [139,140] and apoptin [141]. TRAIL can induce the apoptosis of tumor cells but does not damage normal cells [142]. Zhu *et al*. constructed a TRAIL-expressing OAd, which can efficiently kill triple-negative breast cancer cell lines *in vitro* without damaging normal cells and efficiently inhibit the growth of tumors in animal experiments [139]. Similarly, apoptin can induce the apoptosis of tumor cells but not that of normal cells, and apoptin-expressing OAd can effectively inhibit the growth of gastric carcinoma cells [143].

Conventional chemotherapies lack tumor selectivity and can cause damage to normal cells [144]. By contrast, virus-directed enzyme prodrug therapy is an ideal choice as it involves prodrug-activating enzymes expressed by recombinant OVs to turn nontoxic prodrugs into toxic drugs to specifically kill tumor cells [145]. TK and cytosine deaminase (CD) are widely used prodrug convertases. Chalikonda *et al*. [146] reported on the oncolytic VACV with an inserted CD gene; CD converts the nontoxic prodrug 5-fluorocytosine (5-FC) into 5-fluorouracil (5-FU) in the tumor sites, substantially enhancing the therapeutic effect owing to the presence of active drugs at the tumor sites. However, some tumor cells are resistant to 5-FU, reducing its therapeutic efficiency [147]. TG6002 is an oncolytic VACV expressing the suicide gene FCU1 [148]. FCU1 is a bifunctional gene comprising FCY1 and FUR1. FCY1 can convert 5-FC into 5-FU, and FUR1 can convert 5-FU into the more potent 5-fluorouracil-monophosphate [149], exerting a strong antitumor effect. The results of clinical trials on TG6002 have yet to be reported.

### *ECM-degrading enzymes*

ECM in tumor tissues can inhibit the intratumoral spread of OVs and limit their antitumor efficacy [150]. Many studies have explored the effects of ECM-degrading enzymes, such as relaxin [151], matrix metalloproteinase (MMP)-9 [152], chondroitinase ABC [153], and PH20 hyaluronidase [154]; those ECM-degrading enzymes can increase the intratumoral spread of OVs and improve the antitumor activity of OVs through degrading ECM components. Therefore, expressing ECM-degrading enzymes is a feasible strategy to increase the antitumor activity of modified OVs. For example, GLV-1h255, an oncolytic VACV, can express MMP-9 to promote collagen IV degradation [152] and increase the intratumoral spread and antitumor efficacy of OV. VCN-01 is an OAd expressing PH20 hyaluronidase[155], PH20 hyaluronidase can promote the spread of OVs between tumor cells by degrading the ECM. VCN-01 has now entered clinical trials for the treatment of pancreatic adenocarcinoma and retinoblastoma [156].

### *Anti-vasculature molecules*

Tumor vasculature can transport nutrients and oxygen to tumor cells and is crucial to the growth of tumor cells and an ideal target for antitumor therapy [157]. Previous studies have found that anti-angiogenesis factors expressed by OVs can effectively inhibit tumor angiogenesis and enhance the antitumor efficiency of OVs. Vascular endothelial growth factor (VEGF) can promote angiogenesis, which is an important target of cancer treatment [158]. Alexa *et al*. constructed an oncolytic VACV expressing scAb-targeting VEGF, and its antitumor efficiency was significantly improved [159]. In addition to targeting VEGF, OVs can express other anti-angiogenic factors, such as endostatin [160,161] and thrombospondin-1 (TSP-1), to destroy tumor blood vessels [162]. Endostatin can inhibit tumor angiogenesis by preventing endothelial cell migration; oncolytic HSV expressing endostatin (HSV-Endo) effectively inhibited tumor angiogenesis and growth in the lung cancer model [160]. TSP-1 can inhibit tumor angiogenesis by inducing apoptosis and can improve the antitumor efficiency of OV; T-TSP-1 is an oncolytic HSV expressing TSP-1 that significantly inhibited the growth of gastric cancer *in vivo* [162].

### Monitoring OV replication

Understanding the distribution and replication status of OVs *in vivo* helps in assessing the efficacy and safety of OVs [163]. OV replication can be monitored by incorporating them with reporter genes, such GFP and Rluc (usually used for optical imaging), NIS, and human norepinephrine transporter (NET) (usually used for deep tissue imaging) [164]. GFP insertion is widely used in OVT, and multiple OVs expressing GFP have been developed, such as MV-GFP (MV) [165], JX-GFP (VACV) [104], and rFMW/GFP (NDV) [166]. With the expression of GFP, fluorescence imaging can be used to directly observe the localization of OVs. However, monitoring OVs or tumors in deep tissues via GFP and Rluc is difficult, whereas NIS or NET can be used to monitor OV replication in deep tissues [164]. NIS is a membrane ion channel that mediates iodine transport. After NIS expressed by OVs takes up the iodide isotope, OV localization can be monitored using single-photon emission computed tomography or positron emission tomography [26,167]. MV-NIS is an oncolytic MV expressing NIS, and it has entered clinical trials for the treatment of multiple myeloma [21].

In addition to the insertion of reporter genes, the replication status of OV *in vivo* can be reflected by the expression of soluble markers [168], such as CEA. MV-CEA is an oncolytic MV expressing CEA; the serum CEA level can reflect the replication status of MV *in vivo* [169], but it cannot be used for localization.

## Combination of OVT and other immunotherapies

When OVs are used as monotherapy, their antitumor efficacy can be limited by many factors [186]. Pre-existing antiviral immunity in human bodies could clear OVs rapidly [187]; the heterogeneity of tumor cells might result in the low efficient replication and spread of OVs [187]; the expression of immune checkpoint molecules might limit the antitumor activity of immune cells, which were attracted into the TME by OVs [51]. Researchers have begun to combine OVT with other antitumor therapies, particularly immunotherapies, including ICB (programmed cell death protein 1 (PD-1)/programmed death ligand 1 (PD-L1) and cytotoxic T-lymphocyte-associated protein 4 (CTLA-4) signaling pathway blockade), and CAR-T cell therapies, to further improve the antitumor efficacy of OVs.

### Combinations of OVs and ICB

ICB involves using ICIs to bind to immune checkpoint receptors or ligands and block immune inhibitory signals to treat cancer [188,189]. The PD-1/PD-L1 and CTLA-4 pathways are two important immune inhibitory pathways. Several monoclonal antibodies blocking these two pathways have been approved by the US FDA. The treatment of some tumor types by using ICB is successful [190,191], but many factors that induce resistance of tumor cells to ICB, such as CD8 T cell deficiency in tumor tissues and low expression of immune signal molecules (such as PD-L1), have restricted the antitumor efficacy of ICB [192,193]; many patients also did not benefit from immune checkpoint therapy. Therefore, combinations of OVs with ICB have been widely explored in antitumor research to achieve better outcomes, and some combination therapies have been evaluated in clinical trials (Table 3). The combination of OVs and ICB can exert a synergistic effect. OVs can attract CD8 T cells and natural killer (NK) cells into the TME. This phenomenon addresses the lack of immune cells in the TME and consequent inability of antibodies targeting the PD-1/PD-L1 and CTLA-4 pathways. Anti-PD-1/PD-L1 antibodies can block the PD-L1 induced by OVs (Fig. 3A), and anti-CTLA-4 antibodies can block the corresponding inhibitory signaling pathway and enhance the antitumor activity of immune cells attracted by OVs.

#### *Combinations of OVs and PD-1/PD-L1 blockade*

To date, six monoclonal antibodies targeting the PD-1/PD-L1 pathway have been approved by the US FDA for clinical use; among such antibodies, pembrolizumab, nivolumab, and cemiplimab are anti-PD-1 antibodies [209,210], whereas durvalumab, atezolizumab, and avelumab are anti-PD-L1 antibodies [211]. Tumeh *et al*. [212] analyzed samples from patients with melanoma in the phase I clinical trials of pembrolizumab and found that the CD8 T cell density at the tumor site was significantly lower in patients who did not respond to pembrolizumab

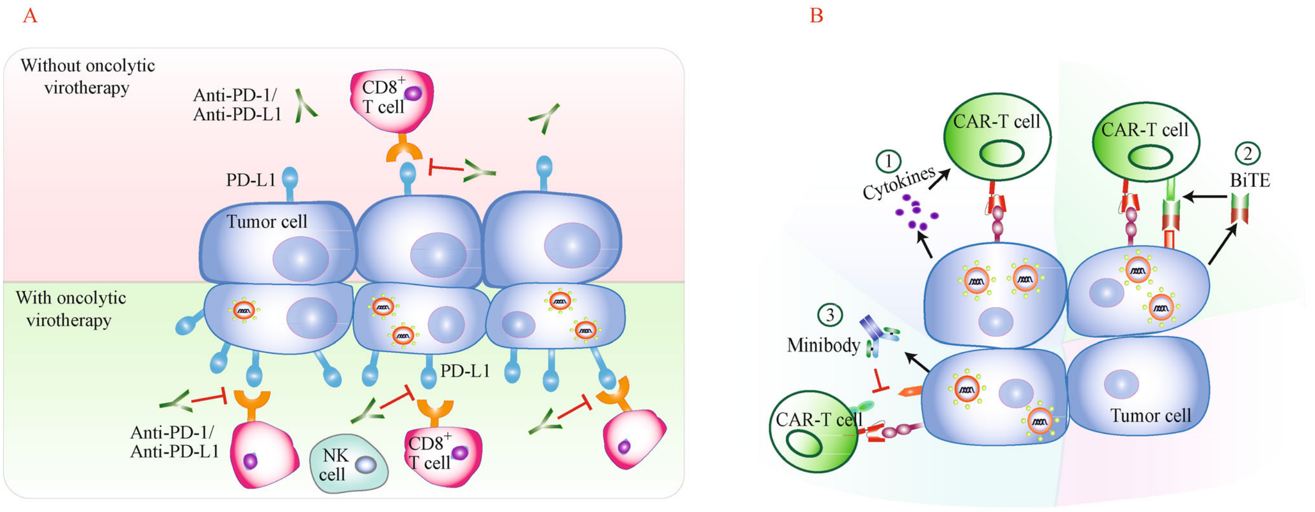

**Fig. 3** Combinations of oncolytic viruses with immune checkpoint inhibitors and CAR-T cell therapy. (A) Mechanisms of OVs combined with ICIs targeting the programmed cell death protein 1 (PD-1)/programmed cell death 1 ligand 1 (PD-L1) pathway. OVs can attract CD8 T cells and NK cells into the TME. The expression of PD-L1 on tumor cells is upregulated by OV infection. Anti-PD-1/PD-L1 antibodies block the PD-1/PD-L1 pathway and stop the immune inhibitory signal. (B) OVs in combination with CAR-T cells in the treatment of solid tumors. Modified OVs can express cytokines that attract CAR-T cells into the TME and enhance their antitumor activities; BiTE-armed OVs express BiTEs that bridge CAR-T cells with tumor cells to prevent the immune evasion of tumor cells; OVs armed with anti-PD1/PD-L1 mini-antibody (mini-body) express anti-PD-1/PD-L1 mini-body blocking the immune inhibitory signal and enhancing the antitumor efficacy of CAR-T cells.

compared with that of patients who responded to pembrolizumab. CD8 T cell deficiency in the TME is an important factor underlying the low efficacy of anti-PD-1 treatment. Studies have reported that OVs can increase the number of CD8 T cells in the TME and peripheral blood [28,213,214], which is helpful in improving the therapeutic effect of PD-1/PD-L1 blockade [189]. OV infections can upregulate PD-L1 expression on the surface of tumor cells [7,194,196,200], while PD-1/PD-L1 blocking antibodies can block the PD-1/PD-L1 signaling pathway. Therefore, combining OVs with PD-1/PD-L1 blockade has synergistic roles in increasing antitumor effects.

Chen *et al*. reported that combining oncolytic HSV-1716 with PD-1 blockade has a pronounced antitumor effect in murine rhabdomyosarcoma models compared with oncolytic HSV-1716 or anti-PD-1 antibody monotherapy; this improvement is associated with an increase in local antitumor T cell immune response [195]. Liu *et al*. combined oncolytic VACV with PD-L1 blocking antibody, which effectively inhibited tumor growth and improved the survival of experimental mice ( $>$ 40% experimental mice were cured) [194] in colon cancer and ovarian cancer models. Fend *et al*. reported that combining the WR strain of VACV with ICB effectively improved the survival of experimental animals with MCA205 sarcoma [199]. Samson *et al*. combined oncolytic reovirus with ICB to treat brain tumors and found that sequential treatment with oncolytic reovirus and PD-1/PD-L1 blocking antibody effectively improved the survival of experimental mice with glioma [7]. Marie-Claude *et al*. also reported that oncolytic Maraba virus can induce long-term antitumor immunity and improve the immunosuppressive status of the TME, enhancing the sensitivity of TNBC to ICB [10]. In the treatment of non-solid tumors, such as acute myeloid leukemia (AML), the combination of OV and PD-1/PD-L1 blockade also produced promising outcomes. Shen *et al*. reported that oncolytic VSV (VSV-IFNβ-NIS) combined with anti-PD-L1 antibodies effectively killed murine AML cells and significantly improved the survival of AML-bearing mice [197].

Combinations of OVs and anti-PD-1/PD-L1 antibodies have been assessed in clinical trials. Ribas *et al*. reported the results of a phase Ib clinical trial of intratumoral T-VEC injection combined with pembrolizumab for the treatment of patients with advanced melanoma [200]. T-VEC enhanced the infiltration of CD4 and CD8 T cells and promoted the PD-L1 expression in tumor tissues, thereby improving the efficacy of pembrolizumab that led to an overall response rate of 62% and a complete response rate of 33%. This combination therapy did not increase toxicity compared with pembrolizumab monotherapy. Sun *et al*. reported the results of a clinical trial of T-VEC combined

**Table 3** Oncolytic viruses combined with immune checkpoint inhibitors and CAR-T cell therapies

| Oncolytic virus | Transgene | ICB/ CAR-T cell | Tumors/tumor cells | Therapeutic outcomes | References |
|---|---|---|---|---|---|
| **Combinations with ICB in preclinical studies** | | | | | |
| vvDD-CXCL11 (VACV) | CXCL11 | Anti-PD-L1 | MC38 colon and ID8 ovarian tumor models | Combination therapy: more than 40% cures in models of colon and ovarian cancers | [194] |
| HSV1716 (HSV-1) | None | Anti-PD-1 | RMS model | Combination therapy significantly prolongs survival of mice bearing M3-9-M tumors | [195] |
| MV-EGFR, MV-NIS (MV) | EGFR-scAb | Anti-PD-1 | GL261 glioma cell | Combination therapy: 60% of mice remained alive for at least 120 days | [196] |
| Reolysin® (Reovirus) | None | Anti-PD-1 | Brain tumor GL261 glioma cell | Combination therapy improves survival | [7] |
| VSV-mIFNβ-NIS (VSV) | mIFNβ, NIS | Anti-PD-L1 | AML | Combination therapy significantly improves the survival of mice with AML | [197] |
| MG1 (Maraba virus) | None | Anti-PD-1 | Human TNBC cell lines, murine mammary carcinoma | Combination therapy significantly slows tumor growth in the 4T1 model | [10] |
| NDV-Fluc (NDV) | Fluc | Anti-CTLA-4 | MC38 colon carcinoma B16-F10 melanoma | More than 80% protection against tumor re-challenge (combination therapy) vs. 40% (anti-CTLA-4 alone) | [9] |
| G47-mIL12 (HSV-1) | IL-12 | Anti-CTLA-4 and anti-PD-1 | Glioma model (CT-2A, 005 GSCs) | Median survival: 66.5 days (triple combination therapy) versus 20 days (mock-treated group) | [198] |
| WR (VACV) | None | Anti-PD1 or anti-CTLA4 | MCA205 tumor | Combination therapy increased survival in MCA205 sarcoma model | [199] |
| **Combinations with ICB in clinical trials** | | | | | |
| T-VEC (HSV-1) | GM-CSF | Pembrolizumab | Advanced melanoma | Overall response rate 62%, complete response rate 33% | [200] |
| T-VEC (HSV-1) | GM-CSF | Pembrolizumab, ipilimumab/nivolumab, or nivolumab | Unresectable stage III–IV melanoma | Overall response rate for on-target lesions: 90% (9/10) | [201] |
| T-VEC (HSV-1) | GM-CSF | Ipilimumab | Unresectable stage IIIB–IV melanoma | Objective response rate: 50% | [202] |
| T-VEC (HSV-1) | GM-CSF | Ipilimumab | Advanced melanoma | Objective response rate: 39% (combination therapy) versus 18% (ipilimumab alone) | [203] |

(*Continued*)

| Oncolytic virus | Transgene | ICB/ CAR-T cell | Tumors/tumor cells | Therapeutic outcomes | References |
|---|---|---|---|---|---|
| **Combinations with CAR-T cell therapies** | | | | | |
| OAd-BiTE (Adenovirus) | EGFR-targeting-BiTE | CAR-T cell targeting the FR-α | Solid tumors (SKOV3, HCT116, and Panc-1 cell) | Median survival: all animals survived until the experimental endpoint of 41 days (CAR-T cells and OAd-BiTE) versus 20 days (OAd-BiTE alone) or 38 days (CAR-T cells alone) | [204] |
| OAd-TNFα-IL2 (Adenovirus) | TNF-α and IL-2 | Mesothelin-redirected CAR-T cell | Pancreatic cancer (BxPC-3, Capan-2, and AsPC-1 cell line, AsPC-1 tumor xenograft model) | Combination therapy efficiently inhibits tumor growth | [205] |
| Ad5Δ24/Ad5Δ24.RANTES.IL15 (Adenovirus) | RANTES and IL-15 | GD2.CAR-T cell | Neuroblastoma cell tumor model | Survival rate: 73% (GD2.CAR-T cells and Ad5Δ24.RANTES.IL-15) vs. 44% (GD2.CAR-T cells and Ad5Δ24) at day 45 | [206] |
| CAd12_PDL1 (Adenovirus) | PD-L1-antibody, IL-12p70 | HER2.CAR-T cell | HNSCC xenograft model | Median survival: more than 100 days (CAd12_PDL1 + CAR-T) vs. 21 or 24 days (control group) | [207] |
| CAd-VECPDL1 (Adenovirus) | PD-L1mini-body | HER2.CAR-T cell | Solid tumor cell lines (PC-3, A549, HepG2) | Median survival: 110 days (twofold longer than mice treated with a single agent) | [208] |

HSV-1, herpes simplex virus-1; VSV, vesicular stomatitis virus; AML, acute myeloid leukemia; GM-CSF, granulocyte-macrophage colony-stimulating factor; TNBC, triple-negative breast cancer; NDV, Newcastle disease virus; RMS, rhabdomyosarcoma; MV, measles virus; BiTE, bispecific T cell engager; EGFR, epidermal growth factor receptor; FR-α, folate receptor α; HNSCC, head and neck squamous cell carcinoma; HER2, human epidermal growth factor receptor 2.

with anti-PD-1 antibody for unresectable stage III–IV melanoma treatment, leading to an overall response rate (on-target lesions) of 90% (9/10) [201].

*Combinations of OVs and CTLA-4 blockade*

Zamarin *et al*. studied the combination of NDV and CTLA-4 blockade in the syngeneic mouse model [9]. The combination generated a synergistic effect, inhibiting the growth of local tumors. In addition, lymphocyte infiltration induced by NDV increased the sensitivity of distal tumor cells (where no OV was injected) to CTLA-4 blockade. Among the CTLA-4 blocking antibodies, ipilimumab has been approved by the US FDA for the treatment of melanoma [215]. In 2018, the results of the phase II clinical trial of T-VEC combined with ipilimumab for melanoma treatment were reported [203], and phase Ib trial results were reported previously [202]. The combination of the two therapies increased antitumor efficacy compared with ipilimumab alone [203] (ORR: 39% versus 18%) with no reduction in safety.

Comprehensive combination regimens have been used for some tumors with a highly immunosuppressed TME. GBM is one of the most lethal tumors, and its TME is highly immunosuppressed. A standalone ICI may not overcome the immunosuppressive state. Saha *et al*. combined anti-PD-1 antibody, anti-CTLA-4 antibody, and oncolytic HSV expressing IL-12 (G47-mIL-12) for GBM treatment [198]. Compared with dual therapy using anti-PD-1 and anti-CTLA-4 antibodies, triple therapy, including anti-PD-1 antibody, anti-CTLA-4 antibody, and G47-mIL-12, cured most experimental mice with 005 GSC GBM and significantly improved the survival of the CT-2A implanted mice. The median survival of CT-2A implanted mice was 66.5, 20, and 19 days for the triple therapy, mock treatment, and dual antibody therapy, respectively. Triple therapy can effectively trigger immune memory and prevent tumor recurrence compared with dual therapy.

**Combinations of OVs and CAR-T cell therapies**

CAR-T cell therapy show considerable promise in cancer treatment. This therapy involves CAR-T cells derived from patients' T cells with an additional insertion of the chimeric antigen receptor (CAR) gene to enhance tumor cell recognition [216]. CAR-T cells targeting CD19 is the first CAR-T cell therapy approved by the US FDA for the treatment of B cell lymphoma [217]. CAR consists of an extracellular antigen recognition domain, a transmembrane region, and an intracellular signal domain [218]. The antigen recognition domain is an antibody fragment that recognizes targeted antigens. The intracellular signaling domain stimulates T cell activation and proliferation to kill target cells [219]. CAR-T cell therapies are successful in the treatment of several hematopoietic malignancies [220,221] but not in the treatment of solid tumors [222]; the immunosuppressive TME is a major obstacle to the role of CAR-T cells [223]. Given the many types of immune-suppressive cells, such as regulatory T cells (Tregs) and myeloid-derived suppressor cells (MDSCs) in the TME [224], and the lack of chemokines to attract tumor-specific T cells, the therapeutic efficacy of CAR-T cell therapy in solid tumors is limited [222].

Combining CAR-T cell therapy with genetically modified OVs can significantly increase CAR-T cell infiltration into the TME and enhance the therapeutic effect of CAR-T cells in solid tumors, as shown in Fig. 3B. Those modified OVs can attract CAR-T cells to the TME via cytokine expression. Nishio *et al*. inserted the genes encoding a chemokine (RANTES) and a cytokine (IL-15) into OAd to create Ad5Δ24.RANTES.IL-15. The intratumoral injection of Ad5Δ24.RANTES.IL-15 increased the infiltration of CAR-T cells targeting the tumor antigen GD2 (GD2. CAR-T cells) into the TME, thereby enhancing their tumor-killing ability [206]. Watanabe *et al*. constructed an AdV expressing TNF-α and IL-2 (OAd-TNFa-IL2) and proved that it could enhance the function of CAR-T cells [205].

Bispecific T cell engager (BiTE) is a fusion protein composed of two scAb fragments; one targets specific molecules on the surface of tumor cells, and the other binds to CD3ϵ on the surface of T cells [225]. BiTE can bridge T cells and tumor cells, thereby promoting T cell activation to kill tumor cells [226]. Blinatumomab (BiTE targeting CD19) has been approved by the US FDA for the treatment of B cell acute lymphoblastic leukemia [227]. The combination of OV expressing BiTE with CAR-T cells can also produce potent antitumor activity [228]. Folate receptor α (FR-α), highly expressed in ovarian, breast, and lung tumor cells, is an important target for antitumor therapy [229]. However, small partial tumor cells remain with low or even no expression of FR-α in those tumor lesions that can lead to immune escape. Wing *et al*. [204] constructed an OAd armed with BiTE targeting epidermal growth factor receptor (OAd-BiTE). In the combination of OAd-BiTE and anti-FR-α CAR-T cells, EGFR-targeting BiTE expressed by OAd bridged the EGFR-overexpressing tumor cells and CAR-T cells to promote CAR-T cell activation and proliferation, and their ability to kill tumor cells was enhanced; as such, the survival of experimental mice was prolonged. All mice treated with CAR-T cells plus OAd-BiTE survived up to the experimental endpoint of 41 days, whereas the median survival of OAd-BiTE-only mice was 20 days and that of CAR-T cells-only mice was 38 days.

Immune inhibitory molecules on the surface of cancer cells also affect the efficiency of CAR-T cell therapy. The transduction of immune checkpoint signals may decrease the therapeutic efficiency of CAR-T cells [230,231], while combinations of ICIs and CAR-T cells can increase the antitumor effects of CAR-T cells [232,233]. Tanoue *et al.* used an OAd expressing anti-PD-L1 mini-antibody (CAd-VECPDL1) to block the transduction of the PD-1/PD-L1 inhibitory signaling pathway and improve the antitumor effect of CAR-T cells; the median survival of mice treated with CAd-VECPDL1 and HER2.CAR-T cells was 110 days, which is twofold longer than that of mice treated with single agent of OAd or CAR-T cells [208]. Compared with the systemic injection of anti-PD-L1 antibody, the anti-PD-L1 mini-antibody (mini-body) produced by OVs mainly localized in the tumor tissue with fewer adverse events [234]. Shaw *et al.* constructed an OAd expressing anti-PD-L1 mini-body and cytokine IL-12p70 (CAd12_PDL1) [207]. IL-12p70 expression could further promote CAR expression and enhance the tumor-killing effect. In the head and neck squamous cell carcinoma (HNSCC) xenograft model, combining CAd12_PDL1 and CAR-T cell therapy improved the median survival time of experimental animals by $>$ 100 days in the CAd12_PDL1 + CAR-T group versus 21 or 24 days in the untreated group. At present, combinations of OVs and CAR-T cells for cancer treatment are still at the preclinical stage and have not yet entered clinical trials (Table 3).

## Conclusions and future prospects

With its development for more than 100 years, OVT has become highly efficient for cancer treatment [3]. OVs can specifically infect and kill tumor cells, remodel the immunosuppressive TME, and stimulate the systemic antitumor response. New viruses with oncolytic potential, such as M1 [75] and ZIKV [115,119], have been continuously discovered, thus further enriching OVT. Genetic modification can improve the safety and antitumor efficacy of OVs, which can be designed to carry different foreign genes to kill different kinds of tumor cells. T-VEC has been approved by the US FDA and EMA for the treatment of melanoma [41]. Some OVs, such as Pexa-Vec and Reolysin®, are being assessed in phase III clinical trials.

The combination of OVT with ICB or CAR-T cell therapies can have synergistic effects, which might play an important role in clinical cancer treatment in the future. OVs can be also combined with other immunotherapies, such as tumor-infiltrating lymphocyte (TIL) therapies [235]. In TIL therapies, patients usually need to be injected with IL-2 to promote TIL proliferation, which might cause many adverse events [236]. The toxicity caused by systematic injections of high-dose IL-2 can be avoided by the intratumoral injection of OAd expressing IL-2 [237]. OVs combined with tumor-targeted monoclonal antibodies, such as trastuzumab [238] and bevacizumab [239] and some small-molecule antitumor compounds, such as trametinib [240] and NU7441 [75], can generate synergistic effects and thus have broad applications in cancer treatment.

However, some shortcomings or problems still confound the full use of OVT. For example, the small genomic capacities of some OVs, such as coxsackievirus and reovirus, limit their ability to accommodate large foreign genes that can optimize their antitumor efficacy [79]. Genetic modification by deleting virulence genes to improve the safety of OVs also reduces the antitumor efficacy of OVs [241]. Although encouraging results and broad applications have been achieved with the combination of OVT and other antitumor immunotherapies, further studies on the combination therapy schedules, including dosages, injection routes, and times, should be conducted to obtain the best antitumor efficiency.

## Acknowledgements

This work was supported by grants from the National Megaprojects of China for Major Infectious Diseases (No. 2018ZX10301403 to LL), the National Natural Science Foundation of China (Nos. 81661128041, 81672019, and 81822045 to LL; No. 81630090 to SJ; No. 81701998 to QW and No. 81703571 to WX), China Postdoctoral Science Foundation (Nos. 2018M640341 and 2019T120302 to SX), and the Sanming Project of Medicine in Shenzhen (to SJ).

## Compliance with ethics guidelines

Qiaoshuai Lan, Shuai Xia, Qian Wang, Wei Xu, Haiyan Huang, Shibo Jiang, and Lu Lu declare no conflict of interest. This manuscript is a review article and does not involve a research protocol requiring approval by relevant institutional review board or ethics committee.

**Open Access** This article is licensed under a Creative Commons Attribution 4.0 International License, which permits use, sharing, adaptation, distribution and reproduction in any medium or format, as long as you give appropriate credit to the original author(s) and the source, provide a link to the Creative Commons licence, and indicate if changes were made.

The images or other third party material in this article are included in the article's Creative Commons licence, unless indicated otherwise in a credit line to the material. If material is not included in the article's Creative Commons licence and your intended use is not permitted by statutory regulation or exceeds the permitted use, you will need to obtain permission directly from the copyright holder.

To view a copy of this licence, visit https://creativecommons.org/licenses/by/4.0/.
